# Diabetes Mellitus is Associated with Poor Bone Microarchitecture in Older Adults Residing in Long-Term Care Facilities

**DOI:** 10.1155/2022/2522014

**Published:** 2022-12-19

**Authors:** Nami Safai Haeri, Mary P. Kotlarczyk, Subashan Perera, Susan L. Greenspan

**Affiliations:** ^1^Division of Geriatric Medicine, Department of Medicine, University of Pittsburgh, Pittsburgh 15213, PA, USA; ^2^Department of Biostatistics, University of Pittsburgh, Pittsburgh 15213, PA, USA

## Abstract

**Objectives:**

Both diabetes mellitus (DM) and osteoporosis are very common in older adults who reside in long-term care (LTC) facilities. Nevertheless, few studies have examined the relationship between diabetes and bone quality in this population. The purpose of this study is to determine if bone mineral density (BMD) or trabecular bone score (TBS) is a better measure of bone quality and skeletal health, in LTC residents with and without a history of DM. *Methodology*. In this longitudinal cohort study, we examined baseline BMD (lumbar spine, total hip, and femoral neck), TBS, DM, and functional status in 511 LTC residents who were enrolled in two ongoing randomized placebo-controlled osteoporosis clinical trials.

**Results:**

On average, participants were older than 80 years and majority were prefrail or frail. Women with DM had greater lumbar spine BMD (1.106 vs 1.017, adjusted difference ± standard error = 0.084 ± 0.023 g/cm^2^, *p* = 0.0003) and femoral neck BMD (0.695 vs 0.651, 0.027 ± 0.013 g/cm^2^, *p* = 0.0463), but lesser lumbar spine TBS (1.211 vs 1.266, −0.036 ± 0.016, *p* = 0.0299) compared to women without DM. Total hip BMD was also higher based on descriptive statistics (0.780 vs 0.734, *p* = 0.6255) in diabetic women, although the difference was not statistically significant. Men had similar but attenuated findings.

**Conclusions:**

Among LTC residents, those with DM have greater BMD but lower bone quality measured by TBS. TBS should be considered in assessing older patients with DM. However, further studies are required to confirm the findings with respect to fractures.

## 1. Introduction

Nearly 1.3 million older adults reside in long-term care (LTC) in the United States. Diabetes mellitus (DM) and osteoporosis are common diseases and major health challenges in LTC residents [1]. The correlation between DM and osteoporosis is well established. Both type 1 diabetes mellitus (T1DM) and type 2 diabetes mellitus (T2DM) are associated with increased risk for osteoporosis and osteoporosis-related fractures in various skeletal sites [2]. Unlike patients with T1DM, the relationship between bone mineral density (BMD) and fracture risk in patients with T2DM is paradoxical. Patients with T2DM generally have increased fracture risk despite a normal or higher than average BMD. Studies that examined possible underlying mechanisms for this paradox identified poor bone microarchitecture as the most likely etiology for low bone strength and increased fracture rate in patients with T2DM with a normal to high BMD [3, 4].

In clinical practice, the quality of bone microarchitecture at the spine can be indirectly measured by trabecular bone score (TBS), that can be derived from the spine dual-energy x-ray absorptiometry (DXA) image. In both diabetic and nondiabetic individuals, decreased TBS correlates with an elevated osteoporotic fracture risk, independent of their BMDs [5]. In patients with T2DM with a normal or elevated BMD, TBS identifies higher percentage of individuals with elevated risk for osteoporotic fractures compared to BMD [6].

The majority of osteoporosis studies with a focus on the association between DM, BMD, and TBS have been conducted in healthy community-dwelling adults [7]. Little data are available on skeletal microarchitecture for adults residing in LTC facilities who are likely to have greater skeletal fragility, poor microarchitecture, lower BMD, and a higher fracture rate, a gap in knowledge we sought to address. We compared BMD and TBS in LTC residents with and without a history of DM who are well characterized for frailty.

## 2. Methods

### 2.1. Study Design

We analyzed baseline patient data from two ongoing randomized placebo-controlled osteoporosis clinical trials in frail LTC residents: ZEST II (Zoledronic acid in frail Elders to STrengthen bone; clinicaltrials.gov registration: NCT02589600) and PROUD (PReventing Osteoporosis Using Denosumab; NCT02753283).

Both the ZEST II and PROUD studies were approved by the University of Pittsburgh institutional review board. Informed consent was obtained from all participants.

### 2.2. Participants

Residents of LTC participating in the ZEST II or PROUD trials were included. Participants were recruited based on BMD, FRAX score, and their fracture history as an adult. All study participants had a diagnosis of osteoporosis, were expected to survive more than 3 years, and were not on dialysis. Participants who did not have any of the above criteria were excluded from the study. Older adults with functional or cognitive impairment were included (Table 1).

### 2.3. Measures

The main outcomes of interest were BMD and TBS.

#### 2.3.1. Bone Mineral Density (BMD)

BMD measured by a DXA scan is currently the standard method for the diagnosis and treatment monitoring in osteoporosis. We measured BMD in total hip, femoral neck, and lumbar spine with Discovery DXA system (Hologic Inc., Bedford, Massachusetts) [8].

#### 2.3.2. Trabecular Bone Score (TBS)

We assessed lumbar spine TBS in patients who underwent DXA scan. Lumbar spine TBS is a gray level textural measurement, extracted from the lumbar spine DXA image. A specialized software (iNsight Software, Medimaps Group, Switzerland) examines pixel variability in the DXA image and, by using a special algorithm, creates a variogram that generates spine TBS value which reflects microarchitecture in lumbar spine [9]. TBS ≥ 1.350 is normal, TBS 1.200 to 1.350 indicates partially degraded microarchitecture, and TBS ≤ 1.200 indicates degraded microarchitecture [10].

#### 2.3.3. Diabetes Mellitus (DM)

The history of DM for each study participant was obtained from a self-reported health questionnaire indicating that the participants' physician has told them that they had diabetes sometime in the past [11].

#### 2.3.4. Activities of Daily Living (ADL)

These are everyday key personal care tasks that are fundamental to maintain full independence at home. Examples include eating, grooming, dressing, ambulating, toileting, and bathing. We assessed ADL by using Katz ADL scale [12]. ADL score ranges between 0 and 14, and higher score indicates more independence.

#### 2.3.5. Instrumental Activities of Daily Living (IADL)

These are more complex tasks that allow an individual to function well in a community. IADL include using phone, preparing meals, taking medication, shopping, managing finances, doing housework, and traveling. We evaluated IADL with Lawton IADL scale [13]. IADL score ranges between 0 and 14, and higher score indicates a higher level of functioning.

#### 2.3.6. Montreal Cognitive Assessment (MoCA)

MoCA is a 12-item screening tool developed by Nasreddine et al. to detect cognitive impairment [14]. Scores 26–30 are considered normal. Lower scores indicate worse cognitive function.

#### 2.3.7. Gait Speed

Gait speed for each patient was calculated by walking time in seconds over a 3 or 4-meter distance. Slow gait speed, particularly speeds slower than 0.6 m/s, has been proven to be a strong predictor for frailty and poor health outcomes [15, 16].

#### 2.3.8. Frailty Category

We used Fried's frailty index and classified participants as nonfrail, prefrail, and frail [17]. In general, frail individuals have consistently higher risk for unfavorable health outcomes and mortality.

### 2.4. Statistical Analysis

All analyses were stratified by gender and were conducted by using SAS® 9.4 software (SAS Institute, Inc., Cary, North Carolina). We used appropriate descriptive statistics to summarize characteristics of participants with and without DM. We used independent samples *t*, chi-square, and Fisher's exact tests to make unadjusted comparisons between those with and without DM. For adjusted comparisons of continuous measures, we used analysis of covariance with age, Duke comorbidity index [11], and smoking history as covariates.

## 3. Results

Of the five hundred and eleven participants, 433 were women and 78 were men (Table 1). Among women, 24% had a history of DM and 56% were prefrail or frail. Average age in women was 80.6 years, BMI was 29.2 kg/m^2^, and gait speed was 0.80 m/s. Among men, 23% had DM and 63% were prefrail or frail. Average age in men was 82.4 years, BMI was 28.7 kg/m^2^, and gait speed was 0.87 m/s. Diabetic women compared to nondiabetics, on average, had a statistically significant higher BMI (31.5 vs 28.5 kg/m^2^, *p* = 0.0003), slower gait speed (0.73 vs 0.82 m/s, *p* = 0.0064), and a lower ADL score (12.6 vs 13.0 points, *p* = 0.0335). There was no statistically significant difference in Fried frailty index, IADL, and MoCA scores between the two groups. Between males with DM and males without DM, no significant difference was recorded with respect to BMI, gait speed, and ADL score.

Women with DM had greater lumbar spine BMD (1.106 vs 1.017, adjusted difference ± standard error = 0.084 ± 0.023 g/cm^2^, *p*=0.0003) and femoral neck BMD (0.695 vs 0.651, 0.027 ± 0.013 g/cm^2^, *p*=0.0463), but lesser lumbar spine TBS (1.211 vs 1.266, −0.038 ± 0.016, *p*=0.0299) compared to women without DM. Total hip BMD was also higher based on descriptive statistics (0.780 vs 0.734, *p*=0.6255) in diabetic women, although the difference was not statistically significant (Table 2).

Based on descriptive statistics, men with DM had higher lumbar spine BMD (1.227 vs 1.208, *p*=0.6861), total hip BMD (0.935 vs 0.894, *p*=0.4848), and femoral neck BMD (0.770 vs 0.728, *p*=0.4559), but lesser lumbar spine TBS (1.255 vs 1.268, *p*=0.7935) compared to men without DM. However, differences were not statistically significant (Table 2).

## 4. Discussion

In frail population of LTC older adults, diabetic women compared with nondiabetics had higher femoral neck and lumbar spine BMD but a lower spine TBS. This is consistent with previous studies that reported similar findings in others who were not residents of LTC. The study by Kim et al. in community-dwelling postmenopausal women and men who were 50 years and older revealed an inverse correlation between lumbar spine TBS and BMD in T2DM patients [18]. In another study by Baleanu et al. in postmenopausal women aged 60–85  years, diabetic women had lower TBS compared with nondiabetics despite similar adjusted BMDs [19]. In our study, total hip BMD was also higher in LTC diabetic women, though it was not statistically significant. Diabetic men in our study had a similar but substantially attenuated pattern in all skeletal sites compared with nondiabetics, with respect to both magnitudes of difference and statistical significance.

The impact of DM on BMD may depend on the subtype of DM and insulin status. In general, patients with T1DM have lower BMDs [20]. This is attributed to low insulin level, high blood glucose (hyperglycemia), and autoimmunity-induced inflammation. Hyperglycemia leads to buildup of excessive amount of reactive oxygen species. Oxidative stress not only is directly toxic to osteoblasts but also interferes with their signaling pathways. Additionally, low insulin status alters substrates essential for osteoblastic differentiation and function [21].

Unlike patients with T1DM, patients with T2DM present with normal or above average BMDs [22]. This is mainly due to bone protective effects of high insulin level as the result of insulin resistance, mechanical tension of excess weight on bones, and elevated leptin levels in T2DM patients with concurrent obesity [23, 24]. Elevated BMD in T2DM limits its utility to assess osteoporosis fracture risk in these patients. Although our data are not sufficiently granular to identify the DM subtype, we speculate that most of our participants with DM had T2DM considering their medical history .

Despite BMD difference in DM subtypes, all diabetic patients have increased incidence of fracture compared to their nondiabetic peers [3]. This is attributed to poor bone microarchitecture and trabecular connectivity along with low bone remodeling speed as the result of DM. Accumulation of advanced glycation end products such as pentosidine in bone matrix and their inhibitory effect on various bone cells, increased marrow fat component, and low bone turnover are some of the possible mechanisms for disrupted bone microarchitecture in DM [25]. DM complications such as neuropathy, increased frequency of falls, and treatment-induced hypoglycemia are also some external factors that contribute to increased fracture risk in these patients [26]. Nonetheless, after adjusting for these external complications including increased fall risk, DM still remains as an independent risk factor for osteoporotic fractures [27].

TBS has been proposed as an index for quality of bone texture and microarchitecture. As a determinant of bone strength, TBS can be used to predict fracture risk independent of BMD [28]. T1DM and T2DM are both associated with poor bone microarchitecture and reduced lumbar TBS [4, 18]. A study by Leslie et al. in community-dwelling women who were 50 years and older showed that lumbar spine TBS compared with BMD is a better predictor of osteoporotic fractures in diabetics [6]. A cross-sectional study by Dhaliwal et al. also revealed that, unlike BMD, TBS is lower in T2DM patients with poor glycemic control irrespective of their age [29].

The main strength of our study is inclusion of frail LTC residents and men. Despite high prevalence of osteoporosis and DM in LTC residents, no study has assessed the effect of DM on TBS in this population to our knowledge. Moreover, we were able to obtain BMD and TBS measures along with frailty and functional status. Most studies exclude frail or functionally impaired participants. In addition, we were able to include a small cohort of men that have been difficult to enroll from LTC facilities since most LTC residents are older women.

Our study also has limitations. First, the history of DM was self-reported. It is unlikely that participants would report this if they did not have diabetes, however, some participants may have diabetes but not realize that they have it or had it in the past. Furthermore, we have no information on the degree, duration, and complications of DM. Another limitation is that most participants in this study are white which may affect generalizability of results. Finally, there were fewer men compared with women that may have limited the ability to see statistically significant differences in men, however, the magnitude of point estimates which are unaffected by sample size suggests an attenuated DM-bone association in men.

## 5. Conclusions and Implications

Our study suggests that in older residents of LTC facilities, DM has greater detrimental effects on bone microarchitecture which may be missed by relying solely on BMD for assessment of bone health. Measures of skeletal architecture such as TBS should be considered in all patients with DM. Further studies are needed to examine TBS and fracture risk prediction in patients with DM.

## Figures and Tables

**Table 1:** Participant characteristics: mean ± standard deviation or *N* (%).

Characteristics	Women (*N* = 433)				Men (*N* = 78)			
	All	DM (*N* = 105)	No DM (*N* = 328)	*p* value	All	DM (*N* = 18)	No DM (*N* = 60)	*p* value
Age	80.6 ± 8.0	71.2 ± 8.0	81.4 ± 7.8	0.0003	82.4 ± 8.3	79.7 ± 8.7	83.3 ± 8.0	0.1110
Race				0.0398				0.0998
Black	31 (7.2)	13 (12.4)	18 (5.5)		2 (2.6)	2 (11.1)	0 (0.0)	
White	401 (92.6)	92 (87.6)	309 (94.2)		74 (94.9)	16 (88.9)	58 (96.7)	
Others	1 (0.2)	0 (0.0)	1 (0.3)		2 (2.6)	0 (0.0)	2 (3.3)	
Frailty				0.3884				0.2049
Nonfrail	190 (43.9)	41 (39.1)	149 (45.4)		29 (37.1)	8 (44.4)	21 (35.0)	
Prefrail	208 (48.0)	53 (50.5)	155 (47.3)		45 (57.7)	8 (44.4)	37 (61.7)	
Frail	35 (8.1)	11 (10.5)	24 (7.3)		4 (5.1)	2 (11.1)	2 (3.3)	
BMI (kg/m^2^)	29.2 ± 6.7	31.5 ± 7.4	28.5 ± 6.4	0.0003	28.7 ± 5.6	29.1 ± 6.2	28.5 ± 5.4	0.7141
Gait speed (m/s)	0.80 ± 0.27	0.73 ± 0.27	0.82 ± 0.26	0.0064	0.87 ± 0.29	0.80 ± 0.30	0.90 ± 0.29	0.2210
ADL (0–14)	12.9 ± 1.4	12.6 ± 1.6	13.0 ± 1.4	0.0335	13.0 ± 1.6	13.0 ± 1.2	13.0 ± 1.8	0.9404
IADL (0–14)	12.5 ± 2.2	12.3 ± 2.2	12.6 ± 2.2	0.1907	12.8 ± 2.0	12.9 ± 2.0	12.8 ± 2.1	0.8492
MoCA (0–30)	24.3 ± 3.9	24.1 ± 4.0	24.4 ± 3.9	0.3870	24.2 ± 3.3	24.4 ± 3.4	24.1 ± 3.3	0.7374

DM = diabetes mellitus, BMI = body mass index, ADL = activities of daily living, IADL = instrumental activities of daily living, and MoCA = Montreal Cognitive Assessment.

**Table 2:** Measures of bone health and diabetes.

Bone measure	Women				Men			
	DM Mean ± SD	No DM Mean ± SD	Adjusted difference ± SE	*p* value	DM Mean ± SD	No DM Mean ± SD	Adjusted difference ± SE	*p* value
BMD (g/cm^2^)								
Lumbar spine	1.106 ± 0.200	1.017 ± 0.204	0.084 ± 0.023	0.0003	1.227 ± 0.197	1.208 ± 0.215	0.023 ± 0.057	0.6851
Total hip	0.780 ± 0.331	0.734 ± 0.235	0.015 ± 0.030	0.6255	0.935 ± 0.182	0.894 ± 0.144	0.029 ± 0.042	0.4848
Femoral neck	0.695 ± 0.123	0.651 ± 0.114	0.027 ± 0.013	0.0463	0.770 ± 0.175	0.728 ± 0.129	0.028 ± 0.038	0.4559

TBS	1.211 ± 0.172	1.266 ± 0.136	−0.036 ± 0.016	0.0299	1.255 ± 0.189	1.268 ± 0.132	−0.011 ± 0.041	0.7935

DM = diabetes mellitus, BMD = bone mineral density, TBS = trabecular bone score, SD = standard deviation, and SE = standard error.

## Data Availability

The data used to support the findings of this study are included within the article.
